# Therapeutic insights elaborating the potential of retinoids in Alzheimer’s disease

**DOI:** 10.3389/fphar.2022.976799

**Published:** 2022-08-23

**Authors:** Tapan Behl, Dapinder Kaur, Aayush Sehgal, Rajeev K. Singla, Hafiz A. Makeen, Mohammed Albratty, Hassan A. Alhazmi, Abdulkarim M. Meraya, Simona Bungau

**Affiliations:** ^1^ School of Health Sciences, University of Petroleum and Energy Studies, Dehradun, Uttarakhand, India; ^2^ Chitkara College of Pharmacy, Chitkara University, Rajpura, Punjab, India; ^3^ Institutes for Sytems Genetics, Frontiers Science Center for Disease-Related Molecular Network, West China Hospital, Sichuan University, Chengdu, Sichuan, China; ^4^ iGlobal Research and Publishing Foundation, New Delhi, India; ^5^ Pharmacy Practice Research Unit, Clinical Pharmacy Department, College of Pharmacy, Jazan University, Jazan, Saudi Arabia; ^6^ Department of Pharmaceutical Chemistry and Pharmacognosy, College of Pharmacy, Jazan University, Jazan, Saudi Arabia; ^7^ Substance Abuse and Toxicology Research Center, Jazan University, Jazan, Saudi Arabia; ^8^ Pharmacy Practice Research Unit, Department of Clinical Pharmacy, College of Pharmacy, Jazan University, Jazan, Saudi Arabia; ^9^ Department of Pharmacy, Faculty of Medicine and Pharmacy, University of Oradea, Oradea, Romania; ^10^ Doctoral School of Biomedical Sciences, University of Oradea, Oradea, Romania

**Keywords:** retinoids, neuroinflammation, RXRS, RARS, neurotransmission, neuroplasticity

## Abstract

Alzheimer’s disease (AD) is perceived with various pathophysiological characteristics such oxidative stress, senile plaques, neuroinflammation, altered neurotransmission immunological changes, neurodegenerative pathways, and age-linked alterations. A great deal of studies even now are carried out for comprehensive understanding of pathological processes of AD, though many agents are in clinical trials for the treatment of AD. Retinoids and retinoic acid receptors (RARs) are pertinent to such attributes of the disease. Retinoids support the proper functioning of the immunological pathways, and are very potent immunomodulators. The nervous system relies heavily on retinoic acid signaling. The disruption of retinoid signaling relates to several pathogenic mechanisms in the normal brain. Retinoids play critical functions in the neuronal organization, differentiation, and axonal growth in the normal functioning of the brain. Disturbed retinoic acid signaling causes inflammatory responses, mitochondrial impairment, oxidative stress, and neurodegeneration, leading to Alzheimer’s disease (AD) progression. Retinoids interfere with the production and release of neuroinflammatory chemokines and cytokines which are located to be activated in the pathogenesis of AD. Also, stimulating nuclear retinoid receptors reduces amyloid aggregation, lowers neurodegeneration, and thus restricts Alzheimer’s disease progression in preclinical studies. We outlined the physiology of retinoids in this review, focusing on their possible neuroprotective actions, which will aid in elucidating the critical function of such receptors in AD pathogenesis.

## Introduction

Alzheimer’s disease (AD) is a progressive neurodegenerative disorder marked with personality changes, memory and cognitive deficits due to loss of the neurons in the frontal cortex and hippocampus. The histological indicator of AD is amyloid plaques, comprising of insoluble amyloid-ß (Aß) peptides (Kabir et al., 2021). Overactive inflammatory astrocytes and microglia localized with the senile plaques are considered to be linked with the pathological lesions (Ayaz et al., 2019a). It is now commonly accepted that mostly affects older individuals over the age of 65 as a geing is a major trigger for Alzheimer’s disease. AD is presently the most common neurological condition affecting over 15 million individuals globally (Andreeva et al., 2017). The world population of individuals with AD is constantly increasing. Clinical evidence links Alzheimer’s disease to dementia and memory loss. AD is characterized by extra-neuronal Aß plaques deposition and intracellular neurofibrillary tangles in the temporal lobe. Aß plaques are made up of accumulated amyloid-beta peptides, whereas neurofibrillary tangles are made up of tau protein that has been hyperphosphorylated (Querfurth and LaFerla, 2010). Oxidative stress, neuroinflammation, and mitochondrial dysfunction are all triggered by the development of these aggregates, resulting in the loss of not only neurons but also white matter in the brain. New research reveals that the pathogenesis of Alzheimer’s disease may be caused by a complicated interaction involving aberrant Aß and tau proteins. The amyloid hypothesis of AD claims that the buildup of Aß plaques in the temporal lobe of the brain is the basic pillar of neurodegeneration and memory deficits in AD patients (Musiek and Holtzman, 2015; Behl et al., 2020). The amyloid hypothesis’s fundamental weakness is its failure to definitively establish the molecular mechanisms that link amyloidosis to NFTsfor neurodegeneration in Alzheimer’s disease (Eriksen and Janus, 2007). There are numerous different possibilities concerning AD pathophysiology, and several natural compounds such as flavanoids, retinoids, lipoic acids have been developed to treat AD based on these beliefs (Ayaz et al., 2019b). The majority of Alzheimer’s models are based on a single theory for the causation of AD, which is a serious flaw in the research. To create viable therapies that will treat the majority of instances, a full understanding of the condition is required. Researchers used different genetically comparable transgenic knock-in animals of AD and tau dysfunction associated with AD and dementia to investigate changes in retinoid signaling at the transcriptional levels in such models. Female rat hippocampal and frontal combined primary cultures were also used to undertake an early assessment of the therapeutic potential of a new family of synthetic retinoids (RAR-M1) that target both biological and non-biological receptors (Endres et al., 2014a; Khatib et al., 2020).

Vitamin-A analogs, both natural and synthetic, are known as retinoids. These chemicals are significant in memory because they are believed to play important functions in adult brain development (Das et al., 2014a). As a result, there is a surge of attention in discovering more about the biology and chemistry of known and new retinoids, as well as their therapeutic potential in the treatment of acute and chronic disorders like AD. Since retinoic acid (RA), a vitamin A derivative, can bind to nuclear receptors and regulate the expression of multiple genes in cells, it executes the majority of biological mechanisms (Lerner et al., 2012). Retinoids activate their target genes by interacting with nuclear receptors including retinoic acid receptors (RAR) and retinoid X receptors (RXR), which are transcriptional modifiers that are reported to be expressed in the prefrontal cortex, amygdala, and hippocampus regions of the brain (Goodman and Pardee, 2003a). These receptors bind to a specific DNA sequence and either suppress or promote target gene expression (Khorasanizadeh and Rastinejad, 2001). In animals, retinoid deprivation or mutations in the RAR and RXR genes have been linked to the suppression of spatial memory and learning, as well as the emergence of depression (Nomoto et al., 2012). In retinoid-deficit rats, suppressing RAR expression resulted in the accumulation of amyloid-beta (Aß) polypeptide in the vasculature, according to research (Shudo et al., 2009). Retinoids play a vital function in neuroprotection by preventing neuroinflammatory processes (Lee et al., 2009a). Microglia, have been shown to suppress the production of neuroinflammatory cytokines and chemokines (Goncalves et al., 2013a). To the improvement of cholinergic neurotransmission, retinoid receptor agonists were known to stimulate the expression of the choline acetyltransferase (ChAT) and acetylcholine transporter genes (Mufson et al., 2008). Retinoids are conventionally accepted as antioxidants, have significant role in the maintenance of brain activity during advanced age. AD patients have been observed with moderate levels of serum vitamin A. Clinically, it was reported that the cognitive abilities in the group of 442 patients of AD were improved with increased serum Vitamin-A levels (Mohammadzadeh Honarvar et al., 2017a). It has been observed that vitamin A and beta-carotene restrain the generation of amyloid-ß fibrils from amyloid precursor protein (APP) and induce variation in the fibrillar anatomy of amyloid beta proteins (Fahrenholz et al., 2010). Retinoic acid (RA), a dynamic metabolite of vitamin A, has been observed to regulate the gene expression relying on APP processing in nuclear receptors including RAR and RXR. Vitamin A deficiency potentiaiates the depoisition of Aß peptides and decreases the long term potentiation of the hippocampus in animals. Researches has revealed that RA potentaite the appearance of the MNSOD2 gene in neuroblast cells, thus decreasing oxidative stress, an essential pathological factor in AD. Studies revealed that memory and cognitive impairments, and reduced neuroplasticity, are due to RXR and RAR mutations (Connor and Sidell, 1997a; Etchamendy et al., 2003a; La Fata et al., 2014; Pierzchalski, 2015).In this review, we discussed the chemistry and biochemistry of various natural and synthetic retinoids, as well as their efficacy in preventing neurodegeneration in Alzheimer’s disease.

## Physiological portrayal of retinoids

**FIGURE 1:**
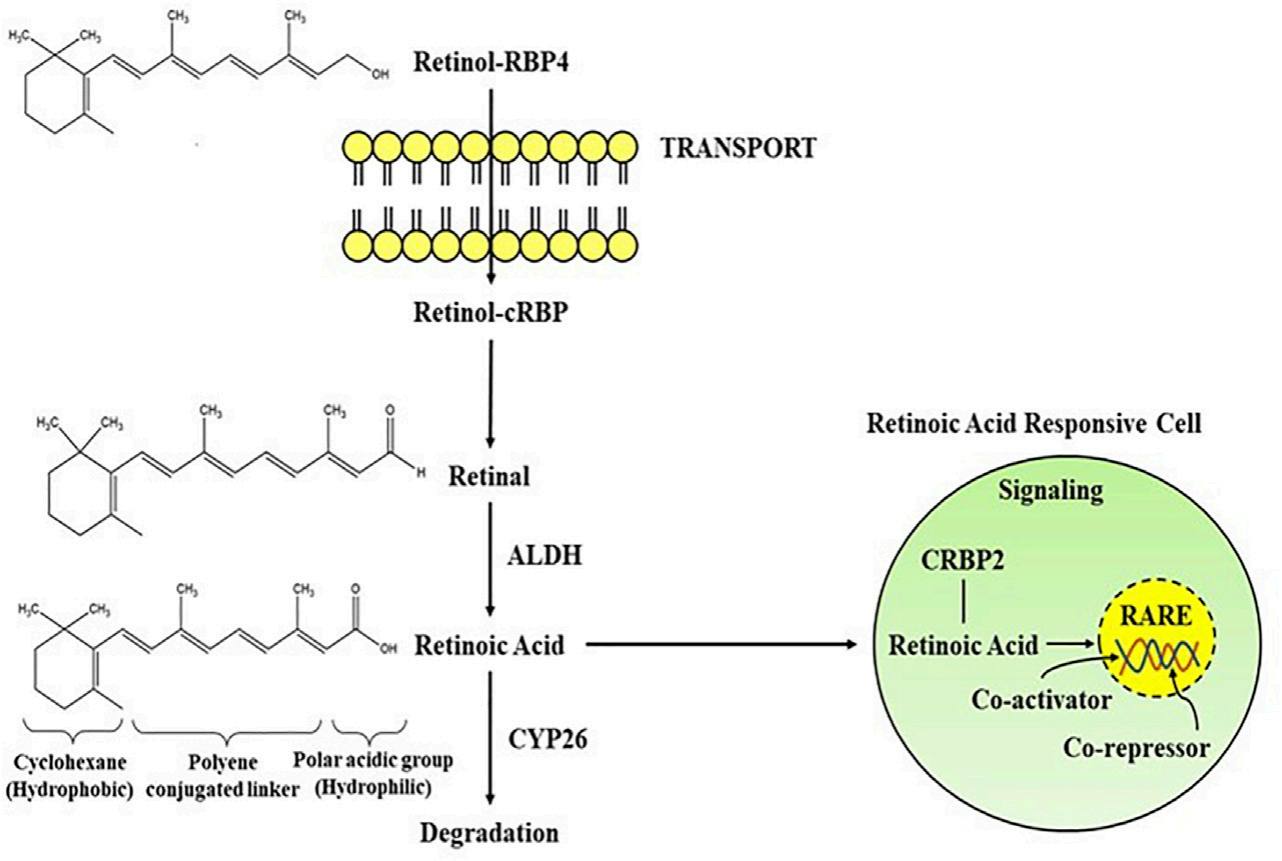
Schematic representation of the transport and signaling pathways of retinoic acid from retinol *via* the activity of alcohol dehydrogenase (ALDH) and degradation through CYP26. Retinoic acid mediates its activity by interacting with the RA receptors in RA responsive cell. RBP, Retinol binding protein; RARE, Retinoic acid response element.

The importance of retinoids in the growth and differentiation of the prenatal and postnatal brain has been recognised long ago (Jiang et al., 2012; Cunningham and Duester, 2015; Bonney et al., 2018). Yet, a substantial size of the data suggests that retinoid signalling is important in adult brain activity as well (Kour and Rath, 2016; Mishra et al., 2018). Vitamin A is the most widely occuring retinoid, regulating a number of physiolgical functions including embryogenesis, cellular proliferation, cell growth, and cell death, as well as proper brain functioning (Khillan, 2014). It is primarily synthesized from pro-vitamin A carotenoids, which may be found in a variety of colourful vegetables and fruits as well as in animal sources like egg yolks, and dairy products. Vitamin A carotenoids are synthesized by some microorganisms and photosynthetic plants which are metabolised to retinol in the small intestines of animals (Green and Fascetti, 2016). Some noteworthy scientific papers have already been published outlining the role of natural and synthetic retinoids in drug development and signaling cascades (Das et al., 2014b; Haffez et al., 2018; Chisholm et al., 2019). At present, there are number of major synthetic retinoids for their therapeutic use, even though some differences in haematological parameters occur following isotretinoin therapy, yet is the most excellent therapy for treating acne vulgaris in clinical studies (Gencoglan et al., 2018). Since, retinoids have conjugated double bonds, they are rapidly oxidised or isomerized by the action of oxidants, light, or intense heat. Increased ingestion of pro-vitamin A carotenoids is linked to a decreased risk of various brain disorders, including Alzheimer’s disease, according to epidemiological research (Lakey-Beitia et al., 2017; Yang et al., 2017). Retinoids also improves visual acuity and protects against age-linked macular degeneration (Harrison, 2019). Therefore, natural and synthetic retinoids are now being explored intensively for effective neuroprotection in neuronal injuries and disorders (Chakrabarti et al., 2016a). The biosynthetic pathway of RA begins either with conversion of retinol to retinal or the manufacture of retinal (Figure 1) as provided by different food sources, often known as vitamin A (Sommer and Vyas, 2012). Two oxidation processes are required to synthesize retinoic acid from retinol. The activity of retinol dehydrogenase and the transition of NAD+ to NADH converts retinol to retinal (Hong et al., 2015). As in the following oxidation reaction, RA is synthesized from retinal with the activity of retinaldehyde dehydrogenases (RALHDs or ALDHs), which come in a variety of types (Kedishvili, 2016). Retinol attach to cellular retinol-binding proteins (CRBPs), whereas RA binds to cellular RA-binding proteins (CRABPs) in the cytoplasm (Napoli, 2017). CRBPs are classified into two classes (CRBP type I and type II); likewise, CRABPs are categorised into two classes (CRABP type I and type II), and they transport RA to RARs and RXRs which are identical but differ in amino acid sequences. CRBPs are essential for the absorption and metabolism of retinol, whereas CRABPs are involved in the control of several RA signalling pathways and the availability of retinol to its receptors (Zhang et al., 2012). All RA isomers activate RARs, which function as heterodimers with RXR nuclear subtypes. Generally, retinoids occur naturally and are chemical conjugates of vitamin A. The RAR-RXR dimer regulates transcriptional activity by interacting with a retinoic acid response element (RARE) found in the gene promoter. Both *α* and *Γ* types of RARs are largely found in the adult brain, with higher expression in both the cortex and the hippocampus, while RARß, RXRΓ, RXRß, and RXR have limited distribution. Although levels might be significant in such confined locations; for example, RAR is expressed primarily in the hypothalamus and striatum. RAR protein’s localized activity does not resemble one of their messenger RNA (mRNA) transcripts, suggesting that post-translational regulation of their representation is important. RA seems to have the ability to control the number of genes actively or passively through RARs (Hong et al., 2015; Kedishvili, 2016). The cytochrome P450 enzymes of the CYP26 family (Napoli, 2017), especially CYP26B1, are principally accountable for shutting down the network (Zhang et al., 2012).

Although the epigenetic roles of retinoids (regulation of transcription) are well-known, the non-genomic function is also important in its physiological activities. The non-genomic activity of retinoids is mostly assumed to be in intracellular pathways, such as stimulation of various kinase pathways, which permits faster indirect effects than the comparatively slow transcriptional processes. RAR is frequently involved in such cytosolic pathways, but retinoids can also interact with some other receptors like CRABP1 or protein kinase-Cα (Ochoa et al., 2003). The modulation of neuroplasticity through stimulation of mammalian target of rapamycin (mTOR) and ERK1/2 phosphorylation-induced mitogen-activated protein kinase (MAPK) is believed to include non-genomic processes (Zhong et al., 2018). RAR’s interaction with the RNA-binding protein PurA is also being hypothesized to prevent axonal protein translation via encouraging mRNA transport to suppress ribonucleoprotein molecules. This inhibition is reversed by retinoids, enabling PurA-mediated axonal RNA transfer to recommence, resulting in neuronal growth stimulation. Aside from intracellular functions, Napoli and Chen found membrane-linked RAR-α in cholinergic neuronal cells, which increases neurogenesis through rapid ATRA-mediated neuronal translation (Chen and Napoli, 2008). Surprisingly, synapto genesis requires the integration of genomic and non-genomic mechanisms (Khatib et al., 2019). Furthermore, specific RAR agonists have different abilities to stimulate genomic and non-genomic systems, which can be engaged separately. RAR-α impacts the cholinergic as well as the GABAnergic and glutamatergic pathways. When RA acts on RAR, it increases the mRNA expression of Choline acetyltransferase (ChAT) and vesicular ACh transporter (VAChT), according to research done on mouse cell lines. As ChAT is necessary for memory encoding and the production of acetylcholine (ACh). Memory encoding and working memory are impaired when muscarinic ACh receptors are blocked, but additional information is enhanced when nicotinic ACh receptors are stimulated (Hasselmo, 2006) (Figure 1).

## Retinoids and neuroinflammation

Dietary supplementation of carotenoids has been shown to play a crucial role in preventing several neu-rodegenerative diseases, including AD (Obulesu et al., 2011a). Retinoids are involved in neuronal patterning, differentiation, and axon outgrowth. Retinoid deprivation leads to impairment of normal brain development and function, resulting in the appearance of symptoms of different neurodegenerative diseases, including AD. Recent investigations indicate that retinoids can induce generation of specific neuronal cell types and also regenerate axons after damage (Maden, 2007). In addition, retinoids are involved in the maintenance of the differentiated state of adult neurons and neural stem cells as well as altered RA signaling levels. Many studies involving genetic analysis of AD have confirmed direct correlation between the genes that encode molecules involved in the RA signaling pathway and those that are considered to be involved in the pathogenesis of AD (Goodman, 2006a).

Retinoids are thought to perform through a variety of processes, according to a substantial amount of research. These chemicals have anti-inflammatory and antioxidant properties. Retinoids have also been discovered to play an important function as antioxidant enzymes as they contribute to the modulation of cytotoxic effects of ROS by eliminating free radicals from cells (Lushchak, 2011). ROS generation and deposition are enhanced in cells during oxidative stress environments like as metal exposure. Retinoids offer protection from this unstable state through a variety of methods, such as inhibition of ROS generation, free radicals clearance, subsequent activation of antioxidant enzymes, and modulation of defence signalling pathways including Nrf2 signalling (Alpsoy et al., 2009). In this view, clinical data suggests that retinoid levels in the liver have been reported as a defence mechanism towards ROS-mediated metal exposure (Defo et al., 2012). Retinoic acid (RA) has been shown to protect neurons from oxidative stress and apoptotic death by lowering glutathione levels (Lee et al., 2009b). In hippocampus cells, it also improves superoxide dimustase (SOD1 and SOD-2) activity (Obulesu et al., 2011b). Furthermore, retinoids are involved in the development and proper functioning of several cells and organs of the immune system, indicating that they play an important regulatory role on various inflammatory responses. The impaired generation of RA-dependent tolerogenic macrophages and dendritic cells in Vit A-deficient cells has been shown to exacerbate inflammatory response (Saurer et al., 2007). Study found that appropriate retinoid levels are required for proper functioning of epithelial barrier stability (Filteau et al., 2001). Retinoids, when consumed in sufficient amounts, have been shown to reduce inflammation in animals (Kang et al., 2007). Also, the anti-inflammatory actions of retinoids involves the inhibition of the synthesis of inflammatory cytokines by reducing the translocation of the NF-kappaB transcription factor (Horton et al., 2001). Additionally, RA has a profound suppressive effect on T-cells (Th cells) which are involved in neuroinflammation whereas RA stimulates Th2, a subtype of T-cell that has anti-inflammatory properties (Nozaki et al., 2006). Furthermore, RA has been demonstrated to increase FoxP3 T-cells, which decrease the inflammation (Kim, 2011). These findings suggest that retinoids are anti-inflammatory agents which may have clinical efficacy in neuroinflammatory disorders including AD (Mohammadzadeh Honarvar et al., 2017b).

Microglia maintain brain homeostasis in normal circumstances. When pathogenic triggers, such as proinflammatory cytokines, pH changes, or hypoxia, are detected, the cells undergo a metamorphosis known as “activation,” which can lead to a chronic prolonged, vicious cycle of “sub-threshold” neuroinflammation. This moderate, yet continually sustained pro-inflammatory condition is thought to represent the neuronal component supporting neurodegenerative pathophysiology (Dantzer et al., 2008). Additionally, cytokines activate indoleamine-2,3-dioxygenase (IDO), a major enzyme in the kynurenine pathway (KP), which destroys the serotonin precursor tryptophan (Hochstrasser et al., 2011) culminating in a microglial-mediated rise in the neurotoxic NMDA-receptor agonist quinolinic acid (Schwarcz et al., 2012). The kynurenine pathway not only serves as a marker for inflammatory activation but also serves as a link between neuro-inflammation and transmitter imbalances, which are linked to a variety of neurodegenerative diseases. As a result, microglial stimulation is a viable therapeutic target for a variety of neurological diseases. Also, Aß causes activation of microglia and austrocytes to produce proinflammatory mediators, while retinoic acid can prevent the development of these proinflammatory cytokines by interacting with RARs, which are found in astrocytic and microglial cells. Retinoids have been shown to stimulate RAR and RXR, allowing these cells to adjust actions and limit production. Among the most fundamental tasks in the therapy of AD is to inhibit neuroinflammatory reactions. Previous research has found that retinoic acid can help in reducing neuroinflammation in neurodegenerative disorders. By reducing the transcriptional activity of the NFK-B, RA was believed to decrease the Aß-mediated generation of TNF-α and suppress the activity of inducible nitric oxide synthase (iNOS) in microglial cells (Kaur et al., 2006). Retinoids also reduce Aß-mediated neuroinflammation, amyloidogenesis, and cognitive impairments in animal models according to the latest research (Behairi et al., 2016). In transgenic mice, RA stimulates neural stem cell growth while suppressing microglial reactivity, resulting in hippocampal neurogenesis (Takamura et al., 2017). In an animal model with Aß-induced neuroinflammation, anti-inflammatory functions of a RAR ligand Am80 (Tamibarotene) (Table 1) were examined, and the analysis indicated that Am80 might endorse the generation of brain-derived neurotrophic factor (BDNF), thus providing cytoprotective outcomes in disease states (Katsuki et al., 2009). Upon administration of Am580 suppressed inflammation-mediated neuronal loss in cultured neurons (Jarvis et al., 2010). In numerous neurological diseases, namely Alzheimer’s disease, RA implies a vital function in suppressing neuroinflammatory reactions and encouraging clearance of Aß additional study is presently being taken for a better comprehension of the microscopic basis for the mechanism of actions of retinoids and carotenoids in the reduction of neuroinflammation in AD. Both retinoic acid and carotenoids provide effective anti-oxidative and anti-inflammatory actions and thus can be used in neuroprotection. They can slow the production and buildup of amyloid plaques, reduce the peroxidation of lipids, and inhibit the release of pro-inflammatory factors, all of which enhance mental abilities (Mohammadzadeh Honarvar et al., 2017c). All such studies have suggested that retinoic acid is performed *via* different routes to provide highly effective neuroprotection in AD. A recent study showed that RA reduced neurotoxicity in the rat brain via regulating the activity of Sirtuin 1, a class 3 histone deacetylase belonging to the Sirtuin protein family, as well as NFκ-B (Priyanka et al., 2018).

**TABLE 1:** The schematic data revealing the potential role of synthetic and natural retinoids as well as vitamin A derivatives in ameliorating the pathogenesis of Alzheimer’s disease in various experimental models through distinct mechanisms/pathways.

S No.	Compound (natural and synthetic)	Biological action through receptor subtype	Potential effects/actions mediated by the corresponding agents	References
1.	Bexarotene	Synthetic RXR Agonist-selectively activates RXRα, RXRß, and RXRΓ subtypes.	• Reverse AB25-35 insulin reduction	Duester (2022)
			• Increase ApoE secretion by RXR activation	
			• Reduce amyloid beta agglomeration in neurons and promotes its clearance from the brain	
			• Improves spatial memory	
2.	Am 80 or Tamibarotene	RXR/RAR Agonist	• Reduces insoluble Aβ40-Aβ42 levels	Maggio and Vlachos (2014)
			• Maintains cortical cholinergic neurotransmission	
			• Reduces anxiety and personality changes	
			• Reduces BACE-1 expression through Nf-kB signaling	
3.	HX 630	RXR Agonist- selectively acts through nuclear receptor subtype RXR and little effect on other subtype	• Improving learning ability	Fukasawa et al. (2012a), Tousi (2015)
			• Reduced microglial activation	
4.	Am 580	RAR Agonist- a stable benzoic acid derivative of retinoic acid which selectively activates RARα isomer	• Reduces Aβ aggregation both intracellularly and extracellularly	Fukasawa et al. (2012b)
5.	All-trans retinoic acid	Retinoic acid isomer	• Downregulation of BACE-1 expression	Kawahara et al. (2014)
			• Modulates NF-kB signaling pathway	
			• Increase IL-10 release	
			• Imrove cognitive and memory abilities	
			• Downregulates NOS production	
6.	Acitretin	Synthetic retinoid	• Increase APPs-a levels in CSF of mild to moderate AD patients	Sodhi and Singh (2014)
			• Decrease production of inflammatory cytokines	
			• Increases expression of ADAM10	
			• Increases IL-6 levels in CSF	
7.	Am 80 + HX 630	RXR/RAR Agonist	• Decrease neuroinflammation and microglial activation	Liu et al. (2015)
			• Production of BDNF	
			• Improve spatial learning	

RA is a CNS morphogen that is recognized to prevent neuroinflammation (Hellmann-Regen et al., 2013) and microglial activation and also, it is important for neuronal growth. RA is a powerful neuroprotective drug that has been linked to neuroplasticity (Maghsoodi et al., 2008). In neurological diseases, each of these pathways is reported to be disturbed. Furthermore, there is a considerable amount of direct evidence supporting retinoid signaling’s role in the pathophysiology of AD (Bremner and McCaffery, 2008) and other neurological diseases (van Neerven et al., 2008), implying that local brain RA might act as an “endogenous antidepressant.” Moreover, the commonly prescribed and well known antidepressant fluoxetine inhibits RA degradation (Wietrzych-Schindler et al., 2011), implying that fluoxetine’s neuroprotective, anti-inflammatory, and perhaps anti-depressant effects are all achieved *via* RA signaling (Hellmann-Regen et al., 2015).

## Retinoids in ameliorating amyloid-ß

**FIGURE 2:**
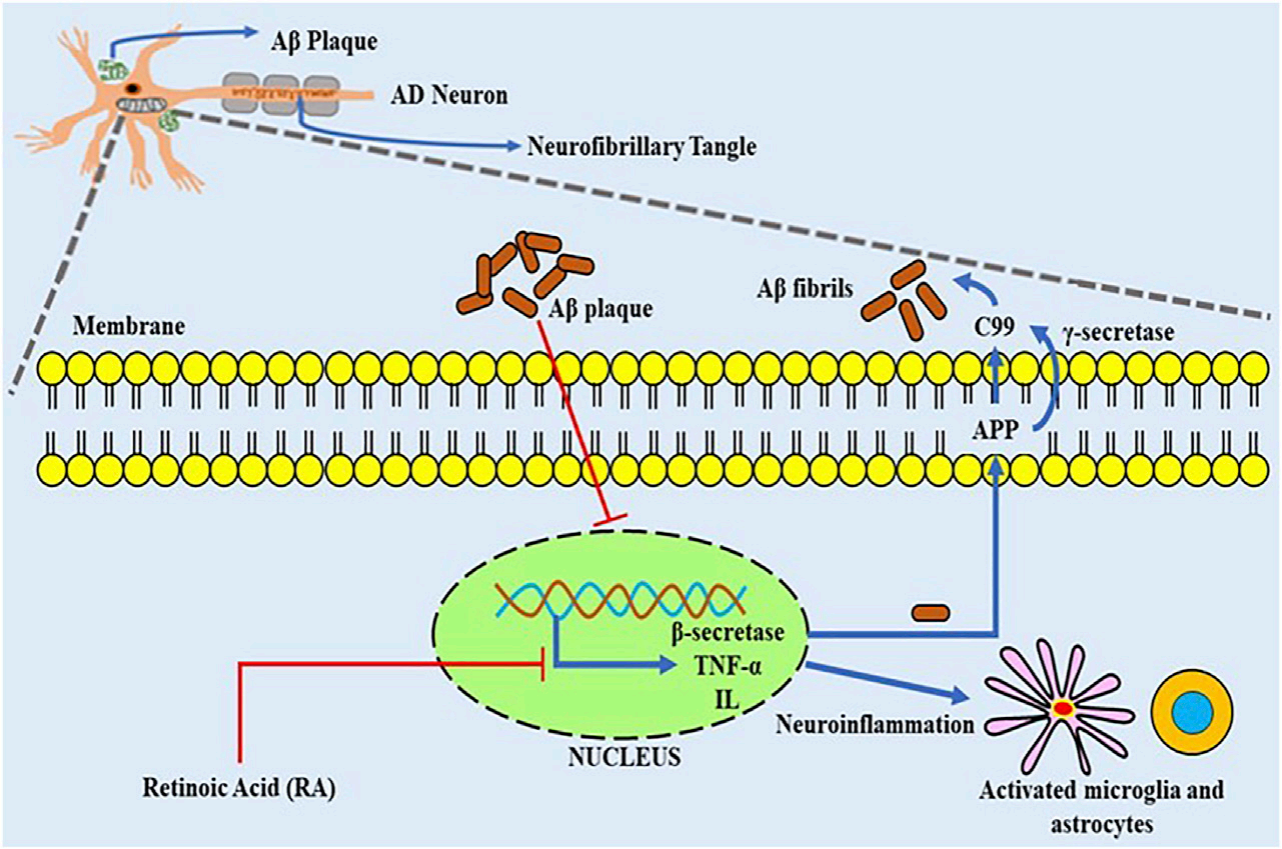
The overactivation of the ß- and Γ-secretases complexes may lead to the formation of Aß fibrils which successively leads to the generation of amyloid-ß plaques and neuroinflammation by activating the pro-inflammatory cytokines like TNF and IL. Such type of overactivation can be inhibited by the activity of retinoids like retinoic acid (RA). TNF-α, Tumour necrosis factor-alpha; IL, interleukins; Aß, amyloid beta; APP, Amyloid precursor protein; C99, carboxyl fragment 99.

Lower intrinsic levels of retinoids have been linked to cognitive loss in the elderly (Huang et al., 2018), and have been found to decrease in the ageing mouse brain (Touyarot et al., 2013). This reduction affects cognition since RA promotes neurogenesis and neuroplasticity, both of which are necessary for memory and learning (Shearer et al., 2012). If low RA levels induce AD, it is reasonable to expect that enhancing them would be beneficial, and multiple *in-vitro* investigations have shown that RA lowers amyloid-beta toxicity (Sahin et al., 2005). Moreover, a vitamin A-deficient diet in mice causes disturbance in RA signalling pathway and Aß accumulation in the blood vessels of frontal brain neurons, which can be restored by RA treatment (Husson et al., 2006a). Increasing the RA signalling through its receptor agonists promotes cognition in transgenic mice models of AD, clears Aß in microglia and neurons, and has a powerful anti-inflammatory effect (Goncalves et al., 2013b). As a result, synthetic retinoids might be used to treat Alzheimer’s disease and other neurodegenerative diseases. Because of its multiple therapeutic benefits, synthetic retinoid tamibarotene (Am80) is being explored intensively as a possible medication for Alzheimer’s disease which tends to reduce the level of insoluble Aß42 in APP23 AD model mice, according (Kawahara et al., 2009) As such acitretin is under investigation, that has been shown to raise the levels of the α-secretase of amyloid-protein precursor (APP), promoting the non-amyloidogenic process in neuroblastoma cells and lowering Aß levels in APP/PS-1 AD model (Tippmann et al., 2009). Acitretin has also been shown to pass the blood-brain barrier in rodents (Holthoewer et al., 2012). Endres et al. (2014b) studied the effects of oral acitretin treatment on α-secretase-acquired APP levels in the cerebrospinal fluid (CSF) of patients with AD. Additionally, acitretin raised APPs- levels and increased non-amyloidogenic processing of APP in humans, according to the studies (Endres et al., 2014b). Although some experimental data on the retinoids utilization in clinical trials is available, research on their possible therapeutic efficacy in AD is still in its initial stages. The data from a clinical trial of bexarotene (RXR ligand) in patients of AD showed that it may decrease cerebral amyloid and enhance plasma Aß1–42 in ApoE4 noncarriers (Cummings et al., 2016). Furthermore, acitretin entered Phase 2 trials in 2010, with preliminary findings showing a 25% rise in APPs-α levels in the CSF in the treatment group of AD patients (Endres et al., 2014c). Additionally, acitretin modulates certain genes connected to the pathogenesis of Alzheimer’s disease, including choline acetyltransferase (chAT). Gonçalves and others tested the effectiveness of numerous retinoid receptor agonists, namely AM 580, CD 2019, and CD437, that are specific RARα, RAR, and RAR *Γ* agonists, accordingly, on the AD experimental model. They showed that activating the RAR receptor signaling pathway promotes Aß removal by increasing the expression of NEP and IDE enzymes and modifying glial cell production of the pro-inflammatory cytokine TNF-α (Kobayashi et al., 1994) (Figure 2).

As a result, a clear link between both reduced IDE mRNA levels and increased Aß deposition was already reported in the AD brain. Furthermore, reducing IDE expression may increase the likelihood of developing AD. Additionally, NEP-associated proteolytic processing of Aß has been shown to activate the signaling of RAR in brain cells (Goncalves et al., 2013a). Therefore, preliminary studies clearly show that natural and artificial retinoids can control Aß production and accumulation, suggesting that retinoids could be employed as a promising therapy for AD pathology as shown in Figure 2. Acitretin, a biosynthetic retinoid, is now being tested in humans under Phase 2 clinical trials. Although, there seems to be no experimental evidence supporting this treatment in Alzheimer’s patients. AD is related to impaired removal of Aß from the brain which is modulated by APOE. IDE is a protein that dissolves Aß deposits and is found in the brain (Manzine et al., 2019). RA regulates IDE transcription, and the gene’s promoter contains a RAR-α response element (Vekrellis et al., 2000). APP is a protein that aids in the production of Aß but is not cytotoxic and stimulates nerve growth (Melino et al., 1996). Also, the MAPT promoter has been demonstrated to be regulated by RA. The examination of MAPT’s (König et al., 1990) nucleotide coding region discovered that it includes a RAR-α response element (Goedert et al., 1989), starting at 5′ -16. The genotype has four distinct TGACC domains, each of which potentially provides retinoid responsiveness (Duester et al., 1991). Variants of one or more RARs may alter the transcriptional activity of such components, according to the latest findings. In contrast to RA, transforming growth factor- ß (TGF-ß), a mediator crucially engaged in AD, Aß plaque generation (Hall et al., 1992), neurological damage, and neuroinflammation, favorably regulates APP translation (Burton et al., 2002). The treatment of TGF-2ß, which decreases amyloid load in mouse models, thus appears to shield terminally differentiated cells encoding TGF-B2 targets against Aß toxicity (Grammas and Ovase, 2002). TGF-2 processes are increased by RA and decreased by their insufficiency (Wyss-Coray et al., 2001). In retinoid-deficit rats, RA restores TGF-ß2 in a tissue-specific manner. As a result, RA may enhance APP synthesis in healthy neurons through the TGF-ß signaling, including SMAD4, which is highly labeled in the AD brain. Transthyretin (TTR) may eliminate or inhibit the production of Aß, according to studies, and greater degrees of this seems to reduce amyloid-induced toxicity (Freemantle et al., 2002). TTR contents were found to be decreased in AD (White and Kelly, 2001), but no TTR alterations were found in an AD sample. Enhanced TTR does have other effects, including stabilizing RARG2 and decreasing the receptor’s responsiveness to RA. The MAPK pathway is inhibited to accomplish this goal. All of the foregoing empirical evidence reinforces the notion that retinoid dysfunction is a critical component tin the emergence of Aß toxicity. This is especially important given the age-related reduction in retinoid availability seen in both healthy and AD humans (Serot et al., 1997). It will be important to analyze these individuals in the coding, 5′, 3′, and promoters to see whether these genetic changes in the retinoid receptors at regions are associated to increases AD susceptibility (Figure 2).

## Retinoids mediated neurotransmission in Alzheimer’s disease

**FIGURE 3:**
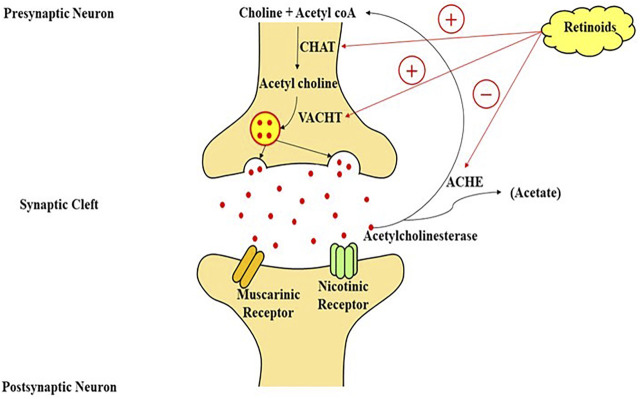
A model of retinoic acid signaling in the central nervous system, which plays an important role in the stimulation of transcriptional machinery to modulate the cholinergic neurotransmission through the production of choline-acetyl transferase mRNA and acetylcholine on the interaction of retinoic acid with its nuclear receptors. CHAT, choline acetyltransferase; VACHT, vesicular acetylcholine transporter; acetyl coA, acetyl coenzyme A; ACHE, acetylcholinesterase enzyme.

Multiple neurotransmitter channels, particularly the catecholaminergic and cholinergic pathways, are disrupted in AD (Mecocci et al., 2002). Cholinergic neurons in the frontal cortex which extends to the neocortex, amygdala, and hippocampus degenerate, which is a characteristic of AD. The depletion of cholinergic neurons causes cognitive issues in experimental animals (Veng et al., 2003; Trillo et al., 2013), and impaired cholinergic signaling is associated with the initial stages of dementia. Treatment of cholinesterase inhibitors (drugs that stop the Ach metabolism and extend its activity in the cortex) was shown to enhance cognition in AD models, and these medications are now being used to address the symptoms of cognitive impairment in patients of neurological diseases (Hunter et al., 2004). Retinoids, which have neurotrophic actions on cholinergic neuronal cells and are markedly reduced in AD patients (Santucci et al., 1989), may serve as a complementary therapy for AD characteristics. RAR stimulation can increase the production of choline acetyltransferase (ChAT) and the VAChT protein, both of these aid in the transportation of Ach into presynaptic vesicles for their release (Goodman, 2006b). Retinoids have been shown to boost Ach and ChAT mRNA levels (Figure 3) (Berse and Blusztajn, 1995a). Dysfunction of monoaminergic pathways, such as the dopaminergic and noradrenergic systems, has also been linked to AD. As a result, the locus coeruleus, a brain’s primary noradrenergic center with considerable distribution in the cortical regions, shows severe deterioration in AD. In AD, tyrosine hydroxylase (a flow-restricting protein for noradrenaline and dopamine production) and dopamine-beta-hydroxylase (DBH, the enzyme essential for noradrenaline generation) levels are also diminished (Ayaz et al., 2021). Furthermore, AD is linked to a lower extent of norepinephrine in the cortex (Mann et al., 1980), and dopamine in the cortex, striatum, and amygdala (Iversen et al., 1983). The trophic effects of RA on the monoaminergic system may help in the mitigation of AD signs because they govern the activity of tyrosine and dopamine hydroxylase (Reinikainen et al., 1988) and could also explicitly regulate the articulation of dopamine D2 receptors by interacting with the RARE promoter site (Pinessi et al., 1987). Some researchers used a delayed recall training test to illustrate the potential usefulness of RA in improving learning and memory. As previously described in regards to RA and AD, this highly utilized scenario for memory is believed to necessitate stimulation of receptors in the 3 neuromodulatory systems: 1) muscarinic cholinergic receptors, 2) dopamine receptors, 3) and adrenergic receptors. The cholinergic receptor antagonist scopolamine was shown to impair memory as predicted, while RAR and RXR agonists were discovered to restore cognitive performance. While the specific process for this impact has yet to be discovered, Shudo and others hypothesized that a retinoid-mediated increase of D2 receptor activation might be one possibility (Kim et al., 2001). Retinoids and forskolin, an adenylate cyclase agonist, had already been demonstrated to enhance the quantity of ChAT mRNA in murine cells. Others have previously found that drugs that elevate cytoplasmic cAMP amount influence ChAT activity transcriptionally in a range of experimental conditions (Samad et al., 1997a; Ledesma and Dotti, 2012; Chakrabarti et al., 2016b). By analyzing the identical blots in sequence with ChAT and VAChT cDNA after a 48-h exposure of the cells with optimum amounts of RA, studies were able to effectively examine the effects of retinoids on ChAT and VAChT mRNA (Walsh and Selkoe, 2007).

Alteration in cholinergic system, which result in a reduction in Ach, are one of the characteristics of AD. The abnormal scopolamine-induced release of Ach due to blocked presynaptic autoreceptor in vitamin A deficient rats, results in cognition loss. Lower synthesis of ChAT, as well as brain cell death induced by RA and the gene promoter, are likely to be the causes of ACh release impairment (Gessel et al., 2012; Saleem et al., 2021). Increased ChAT appears to be favourable, and it has been proposed that RA regulates the vascular ACh transporter as well (Pedersen et al., 1995). In a passive avoidance experiment, retinoid restored scopolamine-derived cognitive loss (Berse and Blusztajn, 1995b). Retinoids also have a role in the expression of tyrosine hydroxylase, dopamine hydroxylase, and the dopamine D2 receptor. Dopamine receptors activity is regulated primarily by RA via interaction with a RA response element (RARE) at the promoter (Shudo et al., 2004).

Hence, retinoid signalling appears to have a function in regulating both acetylcholine production (through ChAT) and cholinergic availability at synapses (*via* AChE) which might serve a function in the pathogenesis and treatment of Alzheimer’s disease. Yet, further research is required (Samad et al., 1997b) (Figure 3).

## Retinoids in potentiating neuroplasticity

**FIGURE 4:**
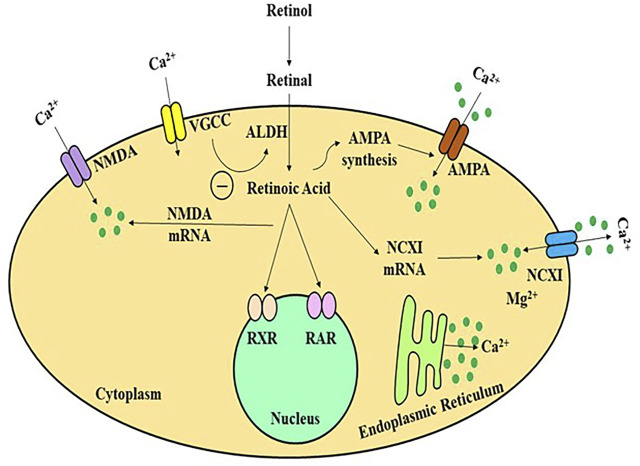
A schematic framework of activity-dependent retinoid generation in neurons. Appropriate synaptic transmission inhibits RA production in a Ca^2+^-dependent manner by activating glutamate receptors and L-type Ca^2+^channels. Limiting glutamate receptors or L-type Ca^2+^ channels reduces dendritic L-type Ca^2+^ intake and de-represses retinoid production, allowing AMPARs to be translated and inserted synaptically neural cells to increase the calcium influx. ALDH, alcohol dehydrogenase; AMPA, amino-hydroxy-methyl-isoxazolepropionic acid receptor; NMDA, N-methyl-D-aspartate receptor; VGCC, volatge gated calcium channel; RAR/RXR, retinoic acid receptors.

Retinol/Vitamin A is a fat-soluble vitamin whose major metabolite, retinoic acid, performs several biological functions comparable to hormones. The hippocampus and adjacent regions produce and metabolise RA, as well as express retinol-binding protein (Lane and Bailey, 2005). The majority of studies on the involvement of retinoids in memory and learning have used Vitamin A deficient (VAD) rats, RA receptor (RAR) transgenic mice, and ageing mouse models, etc. Etchamendy showed that VAD diet in mice model had dramatically decreased spatial memory and learning after 31 weeks, but that after a few days of RA therapy, the behaviour reverted to normal (Misner et al, 2001). VAD-mediated learning and spatial cognition deficits in rats may be facilitated by RA and RARs, according to some investigations (Etchamendy et al., 2003b). RA activates the RAR, a nuclear receptor that controls genes regulating the neuronal proliferation and differentiation, neurite outgrowth, synaptic plasticity, and other activities (Hernández-Pinto et al., 2006; McCaffery et al., 2006). The downstream pathways in RA activation, however, are unknown. Long-term potentiation (LTP) is the most common form of neuroplasticity, which is thought to be the cornerstone of memory and learning. RA levels and RARs, as well as neurogranin (RC3) and neuromodulin (GAP43), which together play a key role in modulating the uptake of Ca^2+^ and Ca/CaMK in neuroplasticity, have all been investigated in analyses of LTP and long-term depression (LTD) and the influence of VAD (Mark et al., 2006). Various *in-vitro* investigations have revealed that NMDA-NR1 may be a promising target for RA in regards to glutamate receptors. In neural development, RA might stimulate NMDA-NR1 subunit expression in human pluripotent adipose tissue stromal cells (Krucker et al., 2002). During growth, the gene expression of NMDA-NR1 can alter dynamically, such that certain quantity of NR1 subunits can be observed throughout all regions of the newborn rats brain, but 3 weeks after birth, this expression appears to reach its peak, before progressively declining to adult human levels (Kulikov et al., 2007). Such arrangement was observed to be comparable with RAR expression in neural tissue in some previous studies (Luo et al., 1996). The link between Ca^2+^, NMDA receptors, and RA signalling *in vivo*, on the other hand, is yet unknown. LTP and LTD, which are the most fundamental processes of cognitive performance, are initiated by Ca^2+^ influx (Jiang et al., 2011). The LTP and LTD were observed to be impaired in RAR or RAR-RXR knockout mice (Bliss et al., 2006). (Figure 4) Furthermore, in cultured hippocampus, acute RA treatment enhances small excitatory post-synaptic activity magnitude (Chiang et al., 1998). The NMDA receptor, as well as other glutamate and non-glutamate receptors (such as TrkB), can control Ca^2+^ influx in neurons (Aoto et al., 2008). Many types of LTP and LTD have been linked to NMDA receptors (Lynch, 2004). The major mechanism for Ca^2+^ influx into neurons through the post-synaptic membrane is thought to be interaction with NMDA receptors (Figure 4) (Isaac et al., 2009). Complex cognitive activities, including memory generation, necessitate RAR activation *via* all-trans-RA (ATRA) to promote neuroplasticity, according to some studies (Fukumori et al., 2010).

Calcineurin is a calcium-binding enzyme that is important for the link between calcium and RA in neuronal cells. Calcineurin suppression promotes the generation of RA. These alterations occur by stimulating the RA generation and RAR stimulation (Etchamendy et al., 2001). RAR is a protein that stimulates the production of calcium-permeable channels in this network. It is nongenomic because it does not involve its traditional job of controlling transcriptional activity, but rather the fast activity of RAR in the cytosol to control protein expression. RAR is continually carried to the nerve cells, where this functions as an mRNA-binding protein, preventing the production of mRNA like glutamate receptor 1 (GluR1), a critical component of the AMPA receptor calcium receptor. When RA interacts with RAR, it causes the receptor to alter the shape and the detachment of GluR1 mRNA, allowing it to be translated (Arendt et al., 2015). The postsynaptic surface is injected with newly translated GluR1 subunit-containing AMPA channels, permitting calcium uptake into the neuron, thus reinforcing the excitatory contact and turning off RA generation, disrupting the neuroplasticity. Thus the loss of neuroplasticity can be cured by shrinking inhibitory synapses as well as the stimulation of excitatory nerve terminals.

The results of this investigation revealed that RA-mediated morphological and physiological neuroplasticity in adult human cortical lines requires mRNA transcription, as evidenced by recent studies on the importance of protein synthesis in neuroplasticity (Poon and Chen, 2008). Synaptopodin was found as a target and regulator of RA-mediated neuroplasticity at the molecular level. Synaptopodin is an actin-regulating protein related to the spinal machinery (Biever et al., 1979), a membrane expansion of the endoplasmic reticulum observed in a subset of the telencephalic dendritic spine. The findings of such studies showed that 1) synaptopodin complexes are noticed in roughly 70% of nerve cells in the cortical region 2) synaptopodin is an indicator of the human spine system; 3) synaptopodin complexes and spine systems are observed in large nerve cells; and 4) a plasticity-mediating signal, such as RA treatment, stimulates the transformation of synaptopodin complexes, spine system. The significance of synaptopodin and the spine system in neuroplasticity is uncertain; although, it has been suggested that they play a role in the local synthesis of protein and regulation of cytosolic calcium dynamics (Deller et al., 2003). Synaptopodin interacts with actin and -actinin and has been proposed to control spinal mobility and long-term spinal integrity *via* Rho-A signaling (Lenz et al., 2021). Synaptopodin and myosin V was discovered recently to be associated with this mechanism (Yap et al., 2020). Synaptopodin might thus be involved in the structural and functional alterations seen at synaptic vesicles undergoing NMDA receptor-induced neuroplasticity (Jedlicka and Deller, 2017). Both retinoids and synaptopodin have already been related to the homeostatic neuroplasticity in mouse neural tissue, as well as the production and transport of Ca^2+^-dependent AMPA receptors. In diseased states of the brain, changes in retinoid signaling and synaptopodin activity have been linked to neuroplasticity abnormalities (Soden and Chen, 2010). As a result, in the neural tissue of patients with AD and cognitive impairment, changes in synaptopodin activity and retinoid signaling have now been found. Considering that retinoids have been suggested as a promising medicinal route for AD-linked cognitive impairment (Endres et al., 2014a). RA may work by altering the expression of synaptopodin and hence enhancing the capability of adult neural cells to enhance neuroplasticity. Eventually, VAD can harm cognitive function throughout postnatal life. One mechanism behind this phenomenon might be VAD-mediated downregulation of neural RAR, which results in lower production of NMDA-NR1 through no direct transcription, impairing neuronal Ca^2+^ excitability and decreasing LTP (Figure 4; Table 1).

## Antioxidative actions of retinoids

Retinoids, vitamin A including carotenoids and beta-carotene, are lipophilic molecules produced by plants and animals which support various processes for cell growth, development, and differentiation (Duester, 2008; Shannon et al., 2017). Vitamin A is ingested in the form of pro-vitamin A carotenoids from plants and preformed vitamin A from animal-derived food which are modified to all-trans-retinol through a reactive pathway in the intestine. Through diet, carotenoids directly acts as ROS scavengers *via* energy transfer (Muller and Bohm, 2011). The deficiency of vitamin A and its metabolites were studied for their coalition with cognitive impairment in adult animals, thus highlighting the importance of sufficient vitamin A levels. Carotenoids are categorized as- 1) Non-pro-vitamin A carotenoids like lutein and 2) pro-vitamin A carotenoids including β-carotene and retinal, and non-pro-vitamin A carotenoids such as lycopene and lutein (Mohammadzadeh Honarvar et al., 2017d). Retinoids perform by various mechanisms and reach out the free radicals in mitochondria, plasma and cell membranes through electron transfer, physical scavenging, and hydrogen abstraction (Woodall et al., 1997). Also, they can react indirectly with several signaling pathways, such as the nuclear factor elytroid-2-related factor 2 (Nrf2), mitogen-activated protein kinase (MAPK), and NF-κB (Palozza et al., 2003; Ben-Dor et al., 2005). The antioxidative effects of retinoids includes quenching of singlet oxygen molecules and scavenging peroxyl radicals. Centrally located retinol-binding proteins (RBPs) are known to control the transport of retinol through the BBB into the brain (MacDonald et al., 1990). Higher levels of retinoids were implicated in the frontal lobe cortex of the postmortem human brain (Craft et al., 2004). ß-carotene, a precursor of retinoic acid, has also been observed in the singlet oxygen scavenging, free radical quenching, and lipid anti-oxidation in plasma. Thus, ß-carotene is considered to be localized in the lipid core of the membranes and implied as one of the favourable antioxidant to scavenge hydrophobic radicals in the membrane for clinical utilization (de Oliveira et al., 2012). Retinoids are structural derivatives of vitamin A which are reported to be involved in normal brain functioning including neuronal growth, development, and differentiation. Retinoids are known to regulate the glucocorticosteroids abundance in the brain which is an essential physiological process that can be observed in various stress-linked conditions to preserve the neuroplasticity within the hippocampus (Bonhomme et al., 2014)**.** Under the oxidative stress conditions including metal injury and ROS accumulation, retinoids preserve the cells against this imbalance through several pathways, such as Nrf-2 and other defensive mechanisms like inhibition of ROS generation, free radical scavenging, induction of antioxidant enzymes. It has also been reported that RA has a neuroprotective role against apoptosis and oxidative stress *via* decreasing glutathione and restoring SOD-1 and SOD-2 in the hippocampus (Ahlemeyer et al., 2001). The role of retinoid signal transduction in the control of dopaminergic neurotransmission was noticed during the high levels of RA-synthesizing enzymes and RAR, which potentially has a significant action on regulation of cell survival, adaptation, and homeostatic regulation of the dopaminergic system (Lévesque and Rouillard, 2007). Retinoid expression plays a significant function in memory and cognitive performance, and synaptic plasticity as well (Crandall et al., 2004). RA supplementation upregulated μ-type opioid receptor 1 (MOR1) and its cascade and alleviated dyskinetic movements, which is a known result of long-term therapy of L-DOPA, in animal model (Pan et al., 2019). Additionally, RA induced the neuroprotective action on dopamine neurons in MPTP-treated mice model of AD. Administration of RA-loaded polymeric nanoparticle largely curbs the dopamine neuron loss in the substantia nigra and axonal innervations in the striatum (Esteves et al., 2015). Moreover, a research on synthetic retinoid revealed the reduction in neuroinflammation, Aß load, and oxidative stress with age-linked cognitive improved in AD patients (Bitarafan et al., 2016).

## Future perspectives

Growing data suggests that retinoid insufficiency may have a role in the pathology of AD, including enhanced Aß accumulation and memory loss (dos Santos Guilherme et al., 2019). Retinoid serum levels were found to be lower in AD patients in a meta-analysis (Das et al., 2019). RA transmission, on the other hand, have been shown to reduce the development of AD in rats (Bonnet et al., 2008; da Silva et al., 2014) and might be used as treatment strategy in patients with AD (Reinhardt et al., 2016). In developing countries, VAD is significantly more common among pregnant women and school—age children (Kitaoka et al., 2013). Vitamin A deficiency may contribute to AD development by disrupting APP processing and resulting in Aß buildup. In AD mouse models, it was discovered that VAD raised Aß levels. Retinoids were implicated in the transcriptional control of the APP and BACE1 genes *via* NF-B and RARs in earlier studies (West, 2002; Endres et al., 2014d). In the mouse model of AD, retinoid deficiency promoted BACE1-induced APP cleavage and Aß production, facilitating senile plaque accumulation as well as cognitive impairments. Additionally, the VAD-induced Aß rise may impede RA production, aggravating AD pathogenesis (Koryakina et al., 2009). A production and clearance are both important factors in controlling Aß levels in the brain. Microglia, a phagocyte and a innate immune cell in the brain, play critical role in removal of Aβ peptides (Husson et al., 2006b). It was found that inhibiting RAR reduced Aß clearance by microglia in some investigation, implying that RAR-dependent Aß breakdown is assisted by microglia (Goncalves et al., 2013c).

*In vitro* data imply that all-trans-retinoic acid may also affect tau protein production, particularly the amount of phosphorylated forms of tau (Lee and Landreth, 2010). The utilization of retinoids with the goal of rectifying or decreasing neurodegenerative effects may also include the regulation of neuroinflammation, another pathological mechanism that causes neuronal and synaptic loss in diseases like AD. *In vitro*, amyloid-ß increases the production and release of the inflammatory cytokines such as tumour necrosis factor- and inducible NOS in microglial cells of AD model. Studies on the clinical benefits of omega-3 fatty acids may actively support the neuroprotective effects of retinoids in dementia. A nutritional investigation of patients with various kinds of dementia revealed that a diet high in antioxidants and polyunsaturated fats, especially vitamin A, may have a preventive role (Zeng et al., 2017). Retinoid signalling has already been proposed as a possible target for developing new Alzheimer’s therapy (Charpentier et al., 1995). Because there is contradictory data in the field, it is difficult to say if AD patients have a dysfunction of RA expression. Rinaldi et al. (2003), Larrieu and Layé (2018) found that serum levels of retinols are lower in AD patients, while Connor and Sidell (Goodman and Pardee, 2003b) found that hippocampus retinoid content is equivalent in AD and control groups. Corcoran et al. (2004), Rinaldi et al. (2003) found lower levels of RAR and RALDH2 in AD brains, indicating that RA signalling is likely to be impaired in patients. Studies in aged mice indicate that RAR levels are reduced which may eventually causes impaired RA-mediated actions. Likewise, biological stimulation of RA signalling can restore the memory and learning as reported in aged animal models (Connor and Sidell, 1997b). However, the pathogenesis of Alzheimer’s disease is complicated, involving many biological activities, and retinoids can influence these systems by influencing expression of genes and serving as an antioxidant. Particular data like “the retinoid activity in AD” should be interpreted with caution. To investigate retinoid treatment for Alzheimer’s disease, researchers must first determine the specific involvement of retinoid and its receptors in the many mechanisms that regulate plaque generation, neurofibrillary tangles, cholinergic signaling, and ApoE activity in the adult brain *in vivo* (Corcoran et al., 2004). It has been proposed that retinoid signalling is a strong, plausible avenue for future Alzheimer’s treatments. It is uncertain if Alzheimer’s patients have retinoid signalling given that RA has been utilised in the treatment of AD and there are contradicting results. For example, GL Fata and others observed the serum level of vitamin A in Alzheimer’s decreased While Connor and Seidel suggested that hippocampal levels of retinoids in Alzheimer’s is similar to that of controls, A decline in RARα concentration and retinal dehydrogenase 2 in Alzheimer’s brain, indicating that retinoid signaling may be impaired in patients, In older mice showed that the frequency of RAR decreased, which may eventually lead to a reduction in retinoic acid-dependent effects. In fact, perceptive deficiencies seen in older mice can be inverted by activating retinoic acid. However, the pathogenesis of Alzheimer’s disease includes numerous and intricate signalling pathways, and retinoids may alter these processes by controlling gene expression in addition to having antioxidant characteristic consideration of vitamin A and its receptors’ effects on numerous pathways, including plaque formation, neuroinflammation, and neurotransmission, is important in order to treat AD with retinoids (Eftekhari et al., 2021; Ayaz et al., 2022).

## Conclusion

Retinoid signaling is clearly involved in neurodegenerative diseases like AD, as well fdas memory and learning. The relevance of sufficient Vitamin A status for such cognitive activities has been highlighted in adult mice and rats, but the exact targeting of retinoid signalling pathways behind these pathology have yet to be identified. Remarkably little is focused on the impact of ATRA signalling on other brain functions, given the enormous number of neuronal functions that could potentially be controlled by retinoids in the adult brain. Transgenic mouse models have revealed a role for retinoids in cholinnergic signaling, gene transcription, neuroplasticity, amyloid plaque formation. Nevertheless, such data may not clearly distinguish between the well-described protective effects of retinoids and neurodegeneration. Future study in this new subject is needed to enhance our knowledge of the role of retinoids in the brain. Retinoids, retinoid ligands, and inhibitors have a lot of promise as treatments for Alzheimer’s, and probably other neurodegenerative disorders. However, we need to learn more about the neuronal genes and molecular pathways that are precisely regulated by retinoids, as well as the physiological consequences in the brain, before such interventions may be explored.
